# Circulating microRNAs from the miR-106a–363 cluster on chromosome X as novel diagnostic biomarkers for breast cancer

**DOI:** 10.1007/s10549-018-4757-3

**Published:** 2018-03-20

**Authors:** Minghui Li, Yan Zhou, Tiansong Xia, Xin Zhou, Zebo Huang, Huo Zhang, Wei Zhu, Qiang Ding, Shui Wang

**Affiliations:** 1 Department of Breast Surgery, First Affiliated Hospital of Nanjing Medical University, 300 Guangzhou Road, Nanjing, 210029 People’s Republic of China; 2 Department of Oncology, First Affiliated Hospital of Nanjing Medical University, 300 Guangzhou Road, Nanjing, 210029 People’s Republic of China; 3 Department of Oncology, Affiliated Hospital of Jiangnan University and the Fourth People’s Hospital of Wuxi, Wuxi, Jiangsu People’s Republic of China; 4 Department of Nursing, Yixing People’s Hospital, Jiangsu, People’s Republic of China; 5 Department of Oncology, Affiliated Jiangsu Shengze Hospital of Nanjing Medical University, No.1399 West Road, Shengze Town, Wujiang District, Suzhou, 215000 People’s Republic of China

**Keywords:** Circulating microRNA, Breast cancer, Diagnosis, Exosomes, qRT-PCR

## Abstract

**Purpose:**

Novel noninvasive biomarkers with high sensitivity and specificity for the diagnosis of breast cancer (BC) are urgently needed in clinics. The aim of this study was to explore whether miRNAs from the miR-106a–363 cluster can be detected in the circulation of BC patients and whether these miRNAs can serve as potential diagnostic biomarkers.

**Methods:**

The expression of 12 miRNAs from the miR-106a–363 cluster was evaluated using qRT-PCR in 400 plasma samples (from 200 BC patients and 200 healthy controls (HCs)) and 406 serum samples (from 204 BC patients and 202 HCs) via a three-phase study. The identified miRNAs were further examined in tissues (32 paired breast tissues), plasma exosomes (from 32 BC patients and 32 HCs), and serum exosomes (from 32 BC patients and 32 HCs).

**Results:**

Upregulated levels of four plasma miRNAs (miR-106a-3p, miR-106a-5p, miR-20b-5p, and miR-92a-2-5p) and four serum miRNAs (miR-106a-5p, miR-19b-3p, miR-20b-5p, and miR-92a-3p) were identified and validated in BC. A plasma 4-miRNA panel and a serum 4-miRNA panel were constructed to discriminate BC patients from HCs. The areas under the receiver-operating characteristic curves of the plasma panel were 0.880, 0.902, and 0.858, and those of the serum panel were 0.910, 0.974, and 0.949 for the training, testing, and external validation phases, respectively. Two overlapping miRNAs (miR-106a-5p and miR-20b-5p) were consistently upregulated in BC tissues. Except for the expression of the plasma-derived exosomal miR-20b-5p, the expression patterns of exosomal miRNAs were concordant between plasma and serum, indicating the potential use of exosomal miRNAs as biomarkers.

**Conclusion:**

We identified four plasma miRNAs and four serum miRNAs from the miR-106a–363 cluster as promising novel biomarkers for the diagnosis of BC.

**Electronic supplementary material:**

The online version of this article (10.1007/s10549-018-4757-3) contains supplementary material, which is available to authorized users.

## Introduction

Breast cancer (BC) is the most commonly diagnosed cancer in females worldwide, accounting for 29% of all new cancers among females in 2015. In addition, BC is the leading cause of cancer death among 20–59-year-old females [1–3]. Improvements in the early detection of BC by mammography, ultrasound, MRI and invasive core needle biopsy have decreased death rates [3]. However, the current diagnostic tools for BC have several limitations. Mammographic screening, which is a gold standard for BC diagnosis, has deficiencies regarding the effects of ionizing radiation and decreased sensitivity for early detection due to increased breast density [4, 5]. Most diagnostic methods require a minimum tumor volume for detection, which can translate into an advanced stage at diagnosis. To date, some circulating tumor markers, such as carcinoembryonic antigen and carbohydrate antigen 15-3, have been used to detect BC, but the sensitivity of these markers is low [6, 7]. Thus, new sensitive, specific, and relatively noninvasive biomarkers that can facilitate the early detection of BC are urgently needed.

MicroRNAs (miRNAs) are a family of small, noncoding RNAs that modulate gene expression at the posttranscriptional level by the promotion of degradation or translational repression of target messenger RNAs (mRNAs) involved in a wide range of important biological and pathological processes [8–11]. MiR-106a–363 is a highly conserved miRNA cluster located on human chromosome X and has two autosomal paralogs, namely, miR-17–92 and miR-106b–25. MiR-17–92, also known as oncomir-1, has been identified as one of the most potent oncogenes because of its amplification and overexpression in a number of malignancies, including diffuse large B-cell lymphoma (DLBCL), Burkitt lymphoma, mantle cell lymphoma, and lung cancer [12–15]. As a paralogous cluster of miR-17–92, miR-106a–363 possibly regulates similar genes and has overlapping functions. In particular, miR-19 and miR-92 are completely identical in both clusters. Recent studies have revealed that miRNAs are stable and easily detectable in serum or plasma and have the potential to be used as biomarkers for the diagnosis, prognosis, and classification of various cancers including BC [16–21]. Several studies have explored the differential expression of circulating miRNAs in BC patients, but very few results are reproducible among laboratories due to population diversity and variations in research methods [22].

In the present study, we focused on the miR-106a–363 cluster, the least-explored paralog of miR-17–92 thus far, which has never been thoroughly investigated in BC. Briefly, we conducted a three-phase study to identify circulating miRNAs for detecting BC based on quantitative reverse transcription polymerase chain reaction (qRT-PCR). Previous reports have not reached a consensus on whether serum or plasma is the superior medium for investigation of miRNAs, and thus, we used both plasma and serum samples. The identified miRNAs were further evaluated in BC tissue samples as well as exosomes isolated separately from plasma and serum. In addition, the potential relationship between the identified miRNAs and clinicopathological features of BC was analyzed.

## Materials and methods

### Patients, samples, and study design

All samples (blood and tissues) were collected from the First Affiliated Hospital of Nanjing Medical University between 2014 and 2016 with approval from the Institutional Ethical Committee and written informed consent. A total of 707 females, including 322 patients with histopathologically confirmed BC and 402 healthy controls (HCs), who underwent routine physical examination at the First Affiliated Hospital of Nanjing Medical University, were enrolled in our study. In total, 400 plasma samples (from 200 BC patients and 200 HCs) and 406 serum samples (from 204 BC patients and 202 HCs) were collected for the study. Among the 290 BC patients, plasma–serum matched samples were obtained from 114 patients. An additional cohort of 32 sample pairs (BC and adjacent normal control tissues) was obtained. Data regarding demographic and clinical characteristics were obtained from our BC database.

Whole blood (5 ml) samples were collected with ethylenediaminetetraacetic acid (EDTA)-containing tubes or SST Advance tubes (BD, New Jersey, USA) from individuals prior to surgery. Plasma and serum samples were separated from blood within 6 h after collection. Plasma was obtained using a two-step protocol [centrifugation at 350 reactive centrifugal force (RCF) for 10 min and at 20,000 RCF for 10 min (Beckman Coulter, USA)], and serum was harvested by centrifugation at 1500 RCF for 10 min and at 12,000 RCF for 2 min after allowing the blood to clot for 30 min. Plasma and serum samples were stored at − 80 °C until required. Tissue specimens were obtained from 32 BC patients without preoperative chemoradiotherapy and stored in liquid nitrogen.

**Fig. 1:**
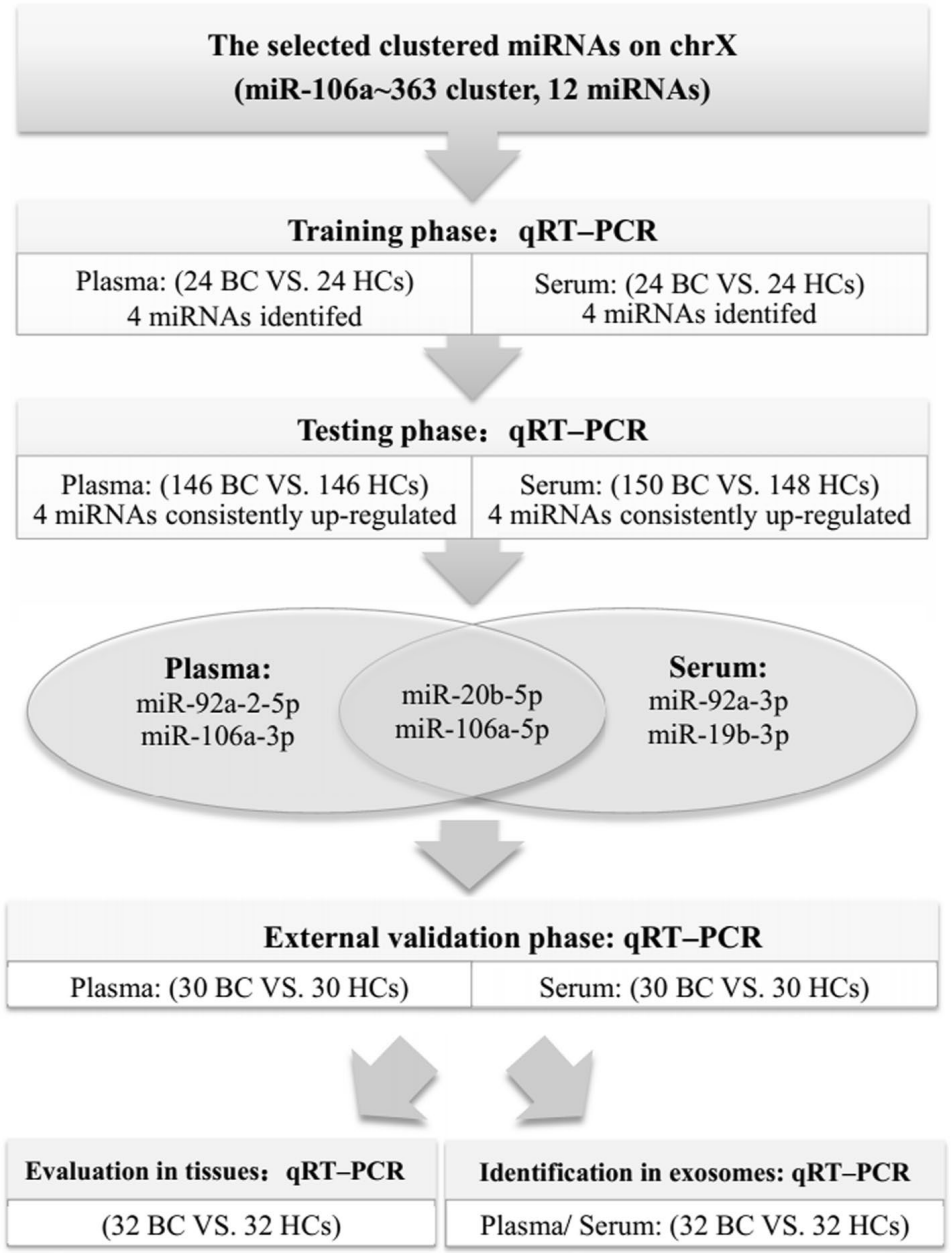
Experimental design

The study was conducted in three phases as shown in Fig. 1. To determine whether the selected clustered miRNAs on chromosome X (miR-106a–363 cluster) are differentially expressed between BC patients and HCs, samples were analyzed by qRT-PCR in the training phase. The significantly differentially expressed miRNAs identified from the training phase were validated in additional samples. To consolidate our findings, the external validation phase was used. In addition, the identified miRNAs were assessed in tissue samples. To explore the potential use of exosome-encapsulated miRNAs as biomarkers, the expression of miRNAs was also determined in separately collected plasma and serum exosomes.

### Classification of epithelial subtypes

Estrogen receptor (ER), progesterone receptor (PR) and human epidermal growth factor receptor 2 (HER2) status was determined by immunohistochemistry (IHC). HER2 status was also assessed through fluorescence in situ hybridization (FISH) if necessary. A tumor was considered to be positive for ER and/or PR if there were at least 1% positive tumor nuclei in the sample [23] and to be positive for HER2 if evaluated as 3+ by IHC or 2+ by IHC with amplified HER2 genes based on FISH [24]. Epithelial subtypes were classified as luminal (ER+  and/or PR+, including luminal A and luminal B), HER2-enriched (ER−, PR− and HER2 positive) and triple-negative (ER−, PR−, HER2−).

### Isolation of exosomes

Exosomes from plasma and serum were isolated using ExoQuick Exosome Precipitation Solution (System Biosciences, Mountain View, CA, USA) as described by the manufacturer. Briefly, 200 µl plasma was pretreated with 2 µl thrombin to obtain a serum-like supernatant and then processed as serum. Exosome pellets were precipitated from a mixture of 200 µl serum and 50 µl ExoQuick Exosome Precipitation Solution, and resuspended in 200 µl RNase-free water for further RNA extraction.

### RNA extraction

Total RNA from 200 µl plasma, serum or exosomes was isolated using the mirVana PARIS Kit (Ambion, Austin, TX, USA) according to the manufacturer’s protocol. Each sample was mixed with denaturing solution (Ambion, Austin, TX, USA) and then spiked with 5 µl synthetic *C. elegans* miRNA cel-miR-39 (5 nM/L, RiboBio, Guangzhou, China) to normalize sample-to-sample variation. TRIzol (Invitrogen, Carlsbad, CA, USA) was used to isolate total RNA from tissue samples according to the manufacturer’s instructions. Total RNA was eluted with 100 μl RNase-free water and stored at − 80 °C until further analysis. RNA concentration and purity were determined using Nanodrop 2000 spectrophotometer (NanoDrop Technologies, Wilmington, DE, USA).

### Quantitative reverse transcription polymerase chain reaction (qRT-PCR)

The expression level of each miRNA was determined using SYBR Green (SYBR^®^ Premix Ex Taq™ II, TaKaRa, Dalian, China). The amplification of an individual miRNA was performed using the Bulge-Loop™ miRNA qRT-PCR Primer Set (RiboBio, Guangzhou, China) containing the specific primers for reverse transcription (RT) and PCR [25, 26]. As previously described, RT reactions were conducted at 42 °C for 60 min followed by 70 °C for 10 min, and qRT-PCR was performed at 95 °C for 20 s, followed by 40 cycles of 95 °C for 10 s, 60 °C for 20 s, and then 70 °C for 10 s on a LightCycler^®^ 480 Real-Time PCR System (Roche Diagnostics, Mannheim, Germany) in 384-well plates [27, 28]. All samples were run in triplicate. The specificity of PCR products was assessed by melting curve analysis. The expression of an individual miRNA from plasma and plasma-derived exosome samples was quantified relative to the expression of the combination of cel-miR-39 (exogenous reference miRNA) and miR-16 (endogenous reference miRNA), while miRNA expression from serum and serum-derived exosome samples was determined relative to the expression of the combination of cel-miR-39 and miR-1228, RNU6B (U6) was used to determine relative expression levels of miRNAs in tissue specimens [29, 30]. The relative expression of each miRNA was determined with the $2^{{ - \Updelta \Updelta C_{\text{t}} }}$ method [31].

### Statistical analysis

To evaluate the stability of expression of plasma and serum miRNAs, GeNorm Version 3.5 was used to calculate the stability value (*M*). To assess the statistical significance of differentially expressed miRNAs in BC, nonparametric tests (Mann–Whitney test) were conducted [32, 33]. The relationship of the identified miRNAs with clinical characteristics was evaluated by one-way ANOVA or *χ*^2^ test, and the correlation of miRNA expression pattern between plasma and serum was analyzed by Pearson’s correlation coefficient (r). Logistic regression analysis was used to establish the miRNA panel. Then, we used receiver-operating characteristic (ROC) curves and area under the ROC curve (AUC) to evaluate the sensitivity and specificity of identified miRNAs or the miRNA panels for BC detection [34].

All statistical analyses and graph plotting were performed using SPSS 20.0 software (SPSS Inc., Chicago, IL, USA) and GraphPad Prism 7.0 (GraphPad Software, USA). P value < 0.05 was set as the level of statistical significance.

## Results

### Characteristics of the study cohort

In total, 400 plasma samples (from 200 BC patients and 200 HCs) and 406 serum samples (from 204 BC patients and 202 HCs) were collected to measure differentially expressed circulating miRNAs within the miR-106a–363 cluster on chromosome X. The plasma and serum samples were separately allocated to three phases: a training phase, a testing phase, and an external validation phase as depicted specifically in Fig. 1. The detailed clinical characteristics of the study participants are given in Table 1 and Additional file 1, and no significant difference was observed in age distribution between BC patients and HCs in any phase (*P* > 0.05).

**Table 1:** Demographic and clinical characteristics of individuals contributing the 400 plasma samples, 406 serum samples, and 32 tissue samples in the study

Characteristics	Plasma samples		Serum samples		Tissue samples
	BC patients	HCs	BC patients	HCs	BC patients
Number	200	200	204	202	32
Age at diagnosis (mean ± SD)	55.11 ± 12.19	50.50 ± 13.59	53.88 ± 11.07	51.10 ± 16.51	51.91 ± 12.89
TNM stage					
In situ	13		12		1
I	54		58		9
II	94		104		15
III	39		30		7
Grade					
I	9		9		2
II	84		66		7
III	107		129		23
Epithelial subtype					
Luminal	107		80		11
HER2-enriched	36		49		10
Triple-negative	44		63		10
In situ	13		12		1

### Selection of endogenous reference

The potential reference miRNAs (miR-16-5p, miR-1228-3p, miR-103a-3p, U6, and miR-191-5p) were examined first, since they have been reported to be highly stable [18, 19, 29, 30, 35]. After analysis by GeNorm, miR-16-5p and miR-191-5p both showed the lowest M value in plasma samples as did miR-1228-3p and miR-103a-3p in serum samples (Additional file 2). Low M values represent small variations in expression and indicate high stability in GeNorm. Based on the combined results from GeNorm analysis and the Ct values (mean ± SD) of each miRNA (plasma miR-16-5p: 25.44 ± 1.44, plasma miR-191-5p: 30.2 ± 2.83, serum miR-1228-3p: 30.24 ± 0.40, and serum miR-103a-3p: 31.36 ± 1.17), miR-16-5p and miR-1228-3p were, therefore, selected as the endogenous reference for plasma and serum, respectively.

### Expression profiles of the clustered miRNAs by qRT-PCR in plasma and serum

**Fig. 2:**
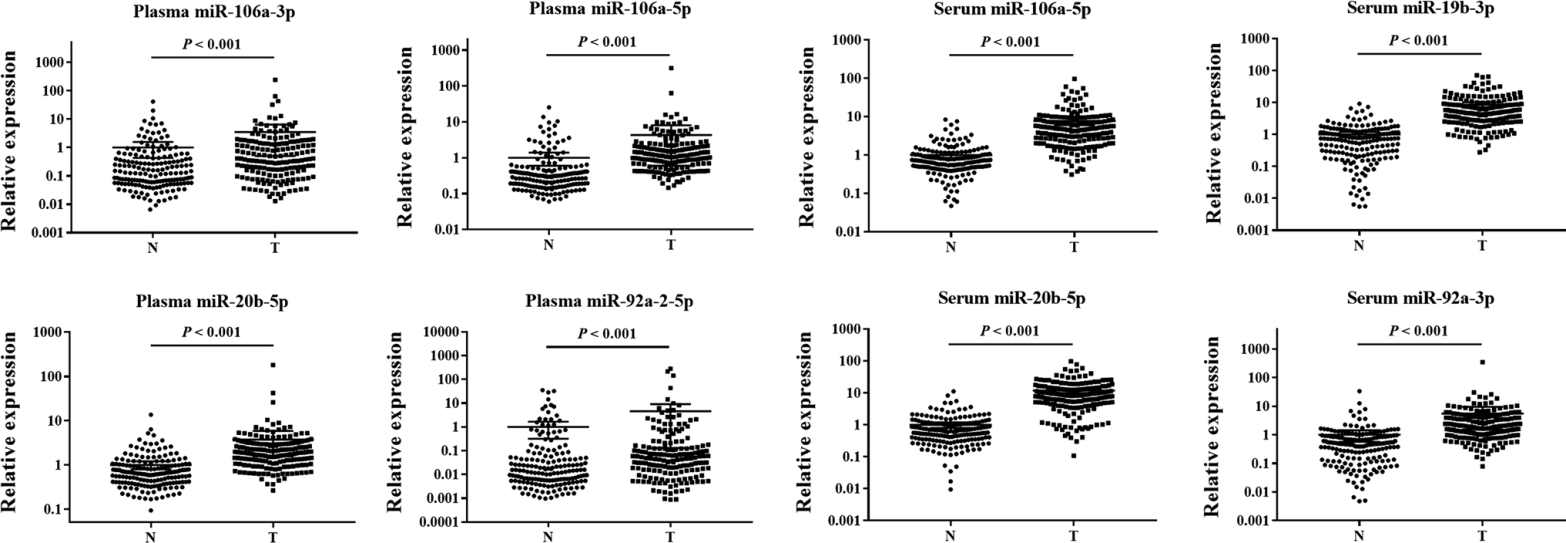
Expression levels of the four indicated miRNAs in plasma samples from 170 BC patients and 170 HCs and the four indicated miRNAs in serum samples from 174 BC patients and 172 HCs (in the training and testing phases). Horizontal line: mean with 95% CI

To identify differentially expressed clustered miRNAs in the plasma of BC patients, we initially investigated the expression levels of 12 miRNAs identified by comparing plasma samples from 24 BC patients and 24 HCs in the training phase. Among these miRNAs, four (miR-106a-3p, miR-106a-5p, miR-20b-5p, and miR-92a-2-5p) were found to be significantly (*P* < 0.05) upregulated in plasma samples and were selected for validation in a larger cohort consisting of samples from 146 BC patients and 146 HCs in the testing phase. All four miRNAs were expressed at higher levels in plasma samples from BC patients than in plasma samples from HCs (Table 2; the other miRNAs are shown in Additional file 3). Furthermore, miR-106a-3p, miR-106a-5p, miR-20b-5p, and miR-92a-2-5p were significantly upregulated in plasma samples from BC patients relative to those from HCs when the results from the training and the testing phase were combined (Table 2, Fig. 2).

**Table 2:** Expression levels of the four plasma miRNAs and four serum miRNAs in the training and testing phases (presented as the mean ± SD; ΔCT, relative to the combination of cel-miR-39 and miR-16 in plasma and cel-miR-39 and miR-1228 in serum; FC: fold change)

MiRNA	Training phase			*P* value	Testing phase			*P* value	Combined	*P* value
	BC	HC	FC		BC	HC	FC		FC	
Plasma										
miR-106a-3p	− 1.50 ± 3.46	0.41 ± 1.67	3.76	0.012	− 2.74 ± 2.38	− 1.48 ± 2.33	2.39	< 0.001	2.55	< 0.001
miR-106a-5p	− 5.71 ± 2.28	− 3.89 ± 1.13	3.54	< 0.001	− 5.95 ± 1.49	− 4.10 ± 1.72	3.60	< 0.001	3.59	< 0.001
miR-20b-5p	− 5.37 ± 1.78	− 3.84 ± 0.90	2.89	< 0.001	− 5.03 ± 1.11	− 3.57 ± 1.16	2.74	< 0.001	2.76	< 0.001
miR-92a-2-5p	− 2.18 ± 3.75	− 0.01 ± 2.49	4.51	0.018	0.06 ± 3.34	1.14 ± 3.48	2.12	< 0.001	2.35	< 0.001
Serum										
miR-106a-5p	− 12.07 ± 1.27	− 10.46 ± 1.77	3.04	0.005	− 14.25 ± 1.34	− 11.25 ± 1.09	8.45	< 0.001	7.02	< 0.001
miR-19b-3p	− 13.99 ± 1.52	− 11.15 ± 3.21	7.14	0.002	− 15.80 ± 1.38	− 12.37 ± 1.76	10.72	< 0.001	10.18	< 0.001
miR-20b-5p	− 10.17 ± 1.38	− 7.62 ± 1.58	5.87	< 0.001	− 13.41 ± 1.27	− 9.77 ± 1.24	13.05	< 0.001	11.25	< 0.001
miR-92a-3p	− 15.71 ± 2.50	− 13.55 ± 2.81	4.46	0.035	− 16.86 ± 1.48	− 14.34 ± 1.92	5.88	< 0.001	5.54	< 0.001

Similar to the procedure used for plasma samples, four consistently upregulated miRNAs (miR-106a-5p, miR-19b-3p, miR-20b-5p, and miR-92a-3p) were identified in serum samples in the training phase and the testing phase (Table 2, Fig. 2). Notably, two miRNAs (miR-106a-5p and miR-20b-5p) showed significantly higher expression levels in BC patients than in HCs in both plasma and serum.

### Diagnostic value of the identified miRNAs in circulation

To evaluate the diagnostic potential of the identified circulating miRNAs in distinguishing BC patients from HCs, we generated ROC curves and calculated AUCs. The AUCs for plasma miR-106a-3p, miR-106a-5p, miR-20b-5p, and miR-92a-2-5p were 0.658 (95% confidence interval (CI) 0.600–0.716), 0.822 (95% CI 0.775–0.869), 0.825 (95% CI 0.780–0.869), and 0.624 (95% CI 0.565–0.684), respectively; and the AUCs for serum miR-106a-5p, miR-19b-3p, miR-20b-5p, and miR-92a-3p were 0.914 (95% CI 0.882–0.945), 0.913 (95% CI 0.884–0.943), 0.915 (95% CI 0.884–0.947), and 0.829 (95% CI 0.786–0.872), respectively, in the combined cohorts from the training and testing phases (Additional file 4).

**Fig. 3:**
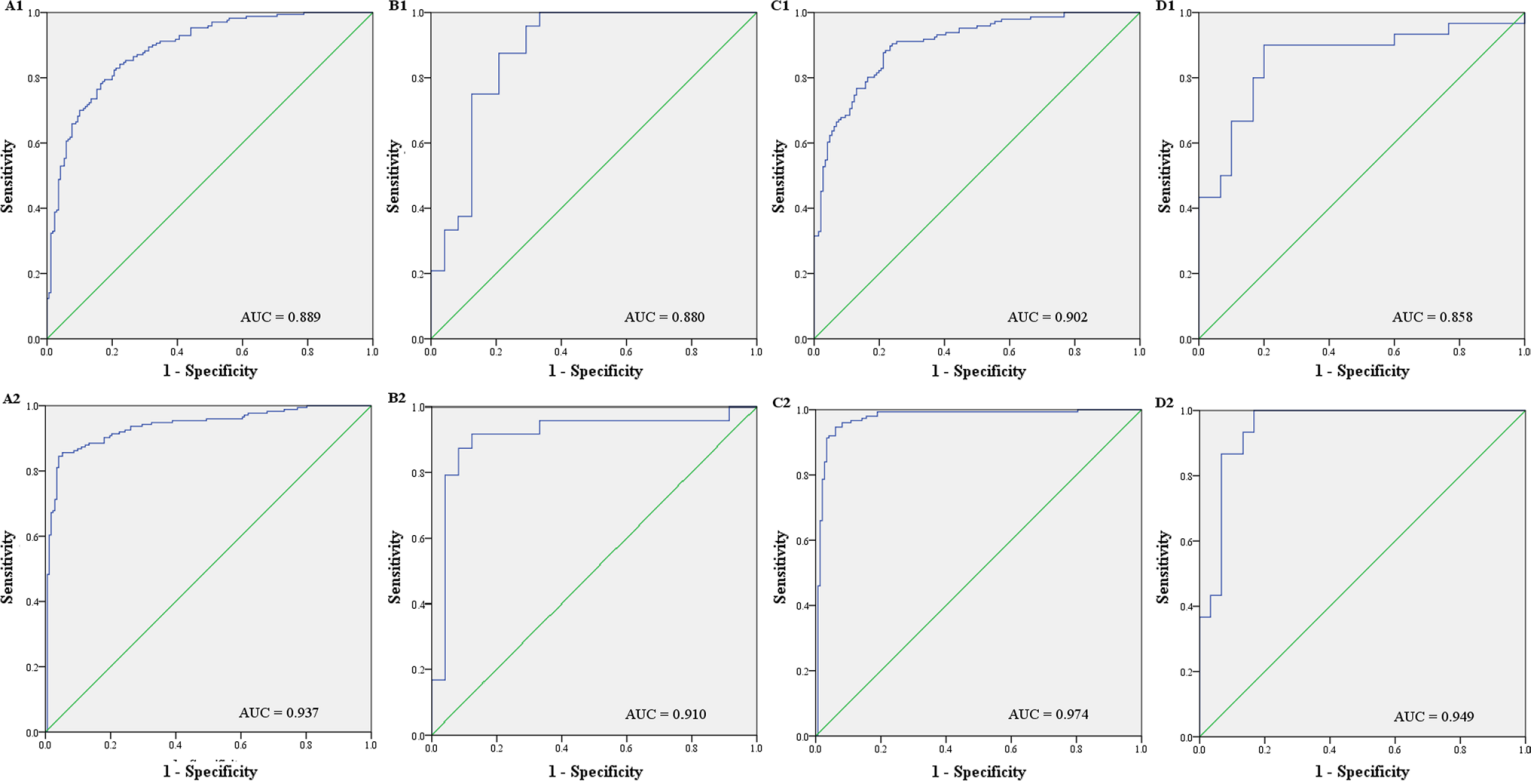
Receiver-operating characteristic (ROC) curves for the plasma and serum 4-miRNA panels for discriminating BC patients from HCs. AUC: area under the curve. **a** Combined two cohorts from the training and testing phases (*A1*: plasma from 170 BC patients and 170 HCs; *A2*: Serum from 174 BC patients and 172 HCs); **b** Training phase (*B1*: plasma from 24 BC patients and 24 HCs; *B2*: Serum from 24 BC patients and 24 HCs); **c** Testing phase (*C1*: plasma from 146 BC patients and 146 HCs; *C2*: Serum from 150 BC patients and 148 HCs); **d** External validation phase (*D1*: plasma from 30 BC patients and 30 HCs; *D2*: Serum from 30 BC patients and 30 HCs)

We then used the four plasma miRNAs and four serum miRNAs to construct two panels. The equation for plasma: Logit(*P*) = − 11.174 + 0.174 × miR-106a-3p − 0.875 × miR-106a-5p − 1.597 × miR-20b-5p + 0.538 × miR-92a-2-5p; and the one for serum: Logit(*P*) = − 15.974 − 0.304 × miR-106a-5p − 1.037 × miR-19b-3p − 0.468 × miR-20b-5p + 0.485 × miR-92a-3p were used to predict probability of BC detection by logistic regression model. Both the plasma 4-miRNA panel and serum 4-miRNA panel exhibited greater performance than any single miRNA in discriminating BC patients from HCs with AUCs of 0.889 (95% CI 0.855–0.923; sensitivity=82%, specificity=79%) and 0.937 (95% CI 0.911–0.964; sensitivity=87%, specificity=89%), respectively, in the combined cohorts (Fig. 3a). The optimal cutoff values were determined at 0.48 for both panels when the data from training and testing phases were combined. We also assessed the diagnostic value of the plasma and serum panels separately in the two phases using the same cutoff values. The AUCs for the plasma panel were 0.880 (95% CI 0.779–0.981; sensitivity=83%, specificity=79%) and 0.902 (95% CI 0.868–0.936; sensitivity=83%, specificity=80%) (Fig. 3B1 and C1), and the AUCs for the serum panel were 0.910 (95% CI 0.811–1.000; sensitivity=91%, specificity=88%) and 0.974 (95% CI 0.955–0.994; sensitivity=94%, specificity=94%) (Fig. 3B2 and C2) for the training and the testing phases, respectively.

Further, an independent cohort of 60 plasma samples (from 30 BC patients and 30 HCs) and 60 serum samples (from 30 BC patients 30 HCs) was employed to separately confirm the diagnostic value of the two panels. The levels of all the identified miRNAs were consistently higher in BC patients than in HCs for both plasma and serum (Additional file 5). The plasma and serum panels could accurately identify BC patients with AUCs of 0.858 (95% CI 0.757–0.958; sensitivity=83%, specificity=80%) and 0.949 (95% CI 0.891–1.000; sensitivity=87%, specificity=87%), respectively (Fig. 3d).

**Fig. 4:**
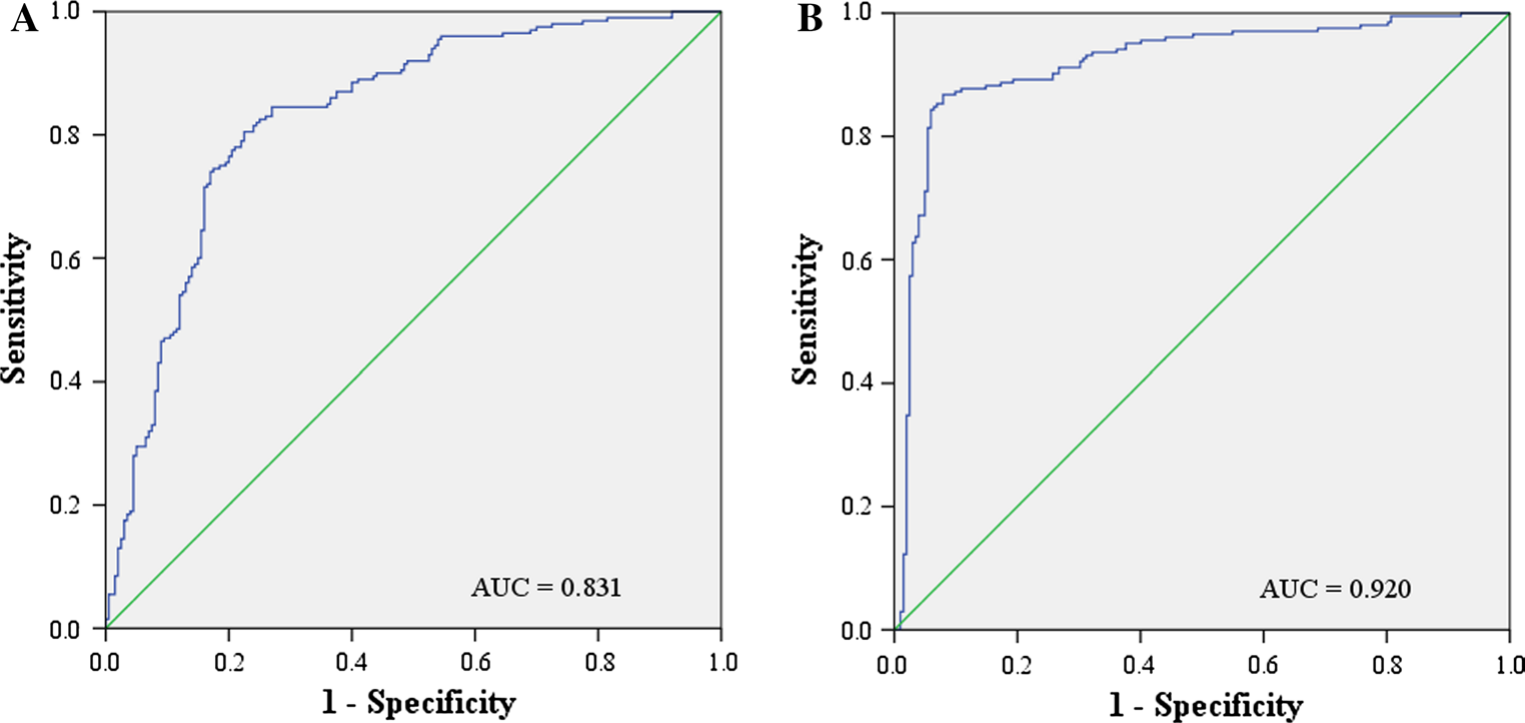
ROC curves for the combination of miR-106a-5p and miR-20b-5p in all plasma and serum samples for discriminating BC patients from HCs. AUC: area under the curve (**a** plasma from 200 BC patients and 200 HCs; **b** serum from 204 BC patients and 202 HCs)

In additional, we investigated the two overlapping miRNAs (miR-106a-5p and miR-20b-5p) in plasma and serum panels. As shown in Table 3 and Fig. 4, the combination of miR-106a-5p and miR-20b-5p also demonstrated favorable diagnostic role in all phases. Correlation analysis was carried out among 114 paired plasma and serum samples from the same patients. Interestingly, the expression levels of miR-106a-5p and miR-20b-5p showed a negative correlation between plasma and serum (Additional file 6).

**Table 3:** Diagnostic performance of the combination of miR-106a-5p and miR-20b-5p for BC using ROC curves and AUC analysis (the optimal cutoff values were 0.47 for plasma and 0.48 for serum)

Phase	Plasma			Serum		
	AUC values (95% CI)	Sensitivity %	Specificity %	AUC values (95% CI)	Sensitivity %	Specificity %
Training phase	0.877 (0.768–0.985)	88	83	0.933 (0.861–1.000)	91	88
Testing phase	0.826 (0.778–0.874)	77	77	0.965 (0.941–0.990)	96	91
Training and testing phases	0.831 (0.791–0.872)	78	78	0.920 (0.890–0.949)	88	89
External validation phase	0.857 (0.757–0.957)	82	83	0.959 (0.894–1.000)	94	88

### Relationship between the identified miRNAs and clinicopathological parameters

We analyzed the association of the identified miRNAs with clinicopathological parameters (TNM stage, histological grade, ER and HER2 status) for the 200 plasma samples and 204 serum samples obtained from BC patients. The levels of all the identified plasma miRNAs (miR-106a-3p, miR-106a-5p, miR-20b-5p, and miR-92a-2-5p) were significantly higher in patients with grade I + II disease than in patients with grade III disease (*P* = 0.024, 0.001, < 0.001 and 0.015, respectively). The levels of plasma miR-106a-5p, miR-20b-5p and miR-92a-2-5p were significantly higher in ER-positive cases than in ER-negative cases (*P* = 0.018, 0.003 and 0.043, respectively). Moreover, higher expression levels of miR-106a-5p and miR-20b-5p were found in HER2-negative cases than in HER2-positive cases (*P* = 0.043 and 0.034, respectively) (Additional file 7). However, no significant relationship was observed between serum miRNAs and characteristics of BC patients (*P* > 0.05). Neither the plasma nor serum miRNAs were significantly associated with clinical TNM stage.

### Evaluation of miRNAs in tissue samples

**Fig. 5:**
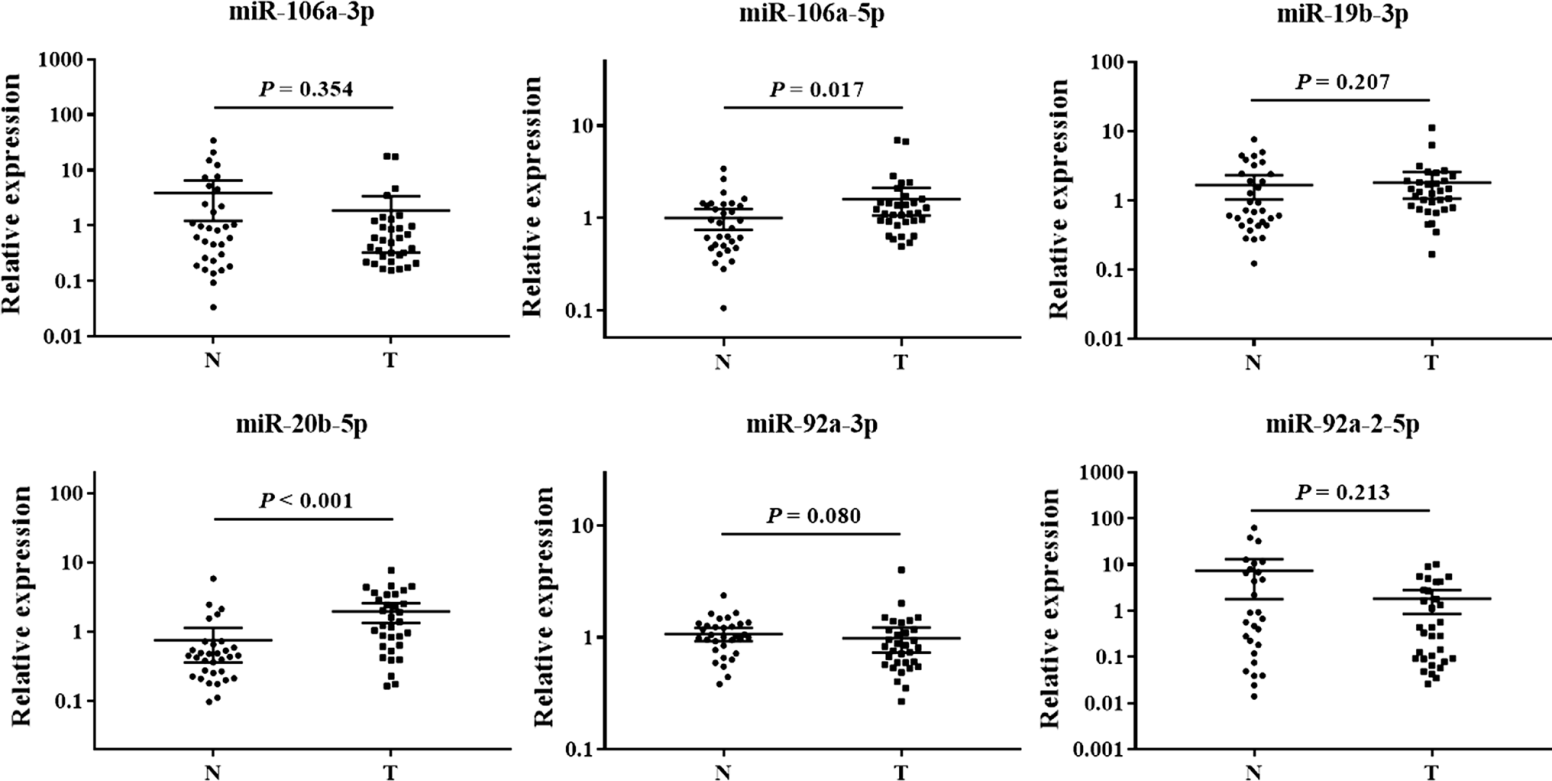
Expression of the 6 identified miRNAs (miR-106a-3p, miR-106a-5p, miR-19b-3p, miR-20b-5p, miR-92a-3p, and miR-92a-2-5p) in the tumor tissues of BC patients. *Y* axis represents the relative expression ($2^{{ - \Updelta \Updelta C_{\text{t}} }}$). Horizontal line: mean with 95% CI. *N* normal control tissue, *T* tumor tissue

To investigate any similarity in elevated expression levels of the 6 identified miRNAs (miR-106a-3p, miR-106a-5p, miR-19b-3p, miR-20b-5p, miR-92a-2-5p and miR-92a-3p) between blood (plasma and serum) and tissue samples, we analyzed 32 pairs of BC and adjacent normal control tissue samples. However, the levels of only the two overlapping miRNAs identified in plasma and serum (miR-106a-5p and miR-20b-5p), but not those of the other four miRNAs, were significantly (*P* < 0.05) higher in BC tissue samples than in adjacent normal tissues (Fig. 5).

### Identification of miRNAs in exosomes

**Fig. 6:**
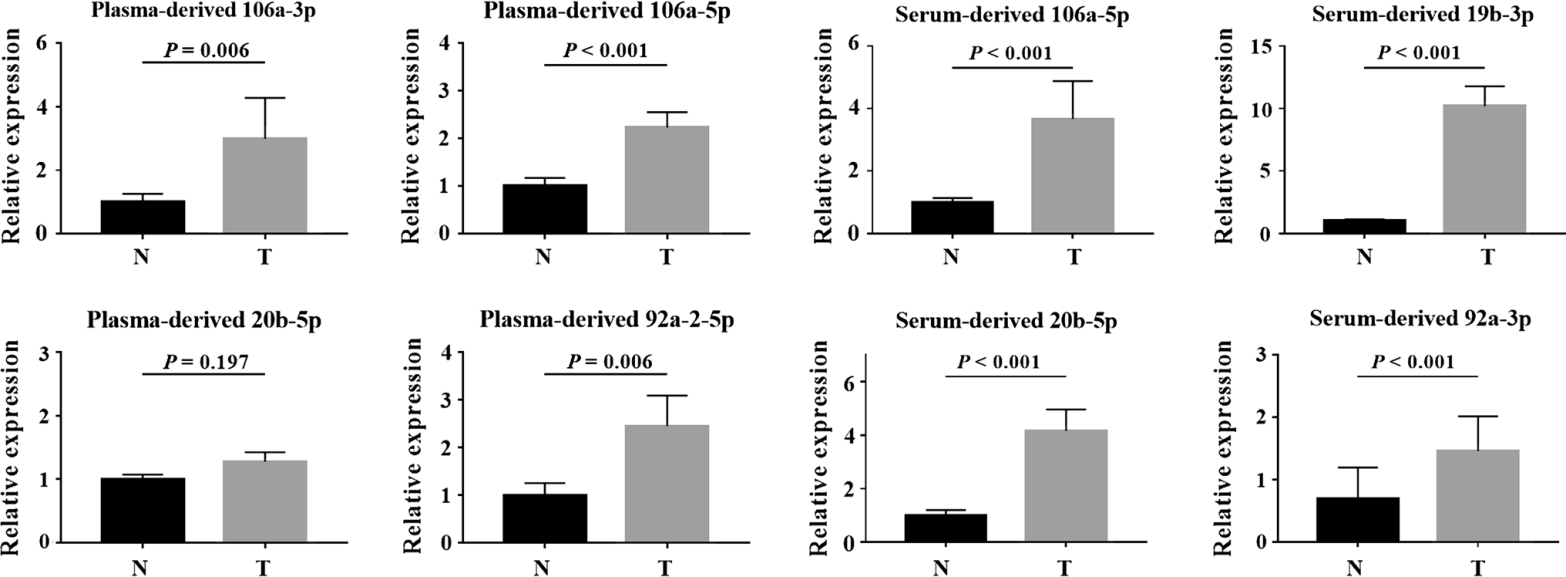
Expression of the identified miRNAs in the plasma and serum exosomes from 32 BC patients and 32 HCs. *Y* axis represents the relative expression ($2^{{ - \Updelta \Updelta C_{\text{t}} }}$). Horizontal line: mean with SEM. *N* normal control tissue, *T* tumor tissue

To explore the potential use of circulating exosome-encapsulated miRNAs as biomarkers for BC, we examined the expression levels of the identified miRNAs in plasma and serum exosomes from 32 BC patients and 32 HCs. Except plasma-derived exosomal miR-20b-5p, all other miRNAs from BC patients exhibited significantly (*P* < 0.05) higher levels than those from HCs (Fig. 6). The results were similar for both plasma and serum.

### Analysis of candidate miRNAs with a bioinformatics database

DIANA-mirPath v3.0 is an miRNA pathway analysis web server for predicting miRNA targets identified using experimentally validated miRNA interactions derived from DIANA-TarBase v7.0. We used this database to decipher the potential function of candidate miRNAs (miR-106a-3p, miR-106a-5p, miR-19b-3p, miR-20b-5p, miR-92a-3p, and miR-92a-2-5p) (Additional file 8) and identified five biological processes “molecular function, cellular protein modification process, enzyme binding, ion binding, and organelle” in gene ontology (GO) category analysis. All but one miRNA (miR-106a-3p) are involved in the “proteoglycans in cancer” pathway in KEGG pathway analysis. Targeted pathways’ heatmaps are shown in Additional file 9. The results indicate the possible roles of the candidate miRNAs in BC pathogenesis.

## Discussion

In this study, we originally focused on the miR-106a–363 cluster, which is located on chromosome X and has never been studied thoroughly in BC despite the strong correlation between sex and the incidence of BC. Our results demonstrated that compared to those in HCs, four plasma miRNAs (miR-106a-3p, miR-106a-5p, miR-20b-5p, and miR-92a-2-5p) and four serum miRNAs (miR-106a-5p, miR-19b-3p, miR-20b-5p, and miR-92a-3p) were significantly upregulated in BC patients, and two panels (plasma and serum) were constructed to detect BC with high sensitivity and specificity. Analysis of the relationship between the identified miRNAs and clinical parameters showed encouraging results in plasma samples.

Previous studies reported several circulating miRNA signatures from either serum or plasma for BC detection [16, 18, 19, 36, 37]. However, there is a limited overlap in significantly differentially expressed miRNAs, and the results of some of those studies are even discordant [22] for various reasons including population diversity, variations in sampling and processing protocols, and differences in analysis methods and endogenous controls. We established a three-phase study and evaluated the expression profiles of the miR-106a–363 cluster using qRT-PCR in both plasma and serum samples from Chinese BC patients and HCs. In the training and testing phases, four plasma miRNAs and four serum miRNAs were found to be significantly upregulated in BC patients. A single miRNA might have potential roles in several diseases; however, a panel may have a higher specificity [38]. Subsequently, the plasma 4-miRNA panel and serum 4-miRNA panel were applied to discriminate BC patients from HCs, and both panels exhibited great performance. The external validation phase was employed to verify the reproducibility and reliability of the diagnostic value of the two panels. Because of the similar expression patterns of plasma miR-16-5p and serum miR-1228-3p in BC patients and HCs in our study, based on the results from GeNorm analysis and the Ct values of each miRNA, miR-16-5p and miR-1228-3p were used as the endogenous reference for plasma and serum samples, respectively. In addition, the identified miRNAs were investigated in BC tissues and exosomes.

We assessed plasma samples from 200 BC patients and 200 HCs and serum samples from 204 BC patients 202 HCs, including 114 matched plasma–serum samples. This is by far the largest cohort study of circulating miRNA signatures in both plasma and serum from BC patients. We identified some differences in miRNA expression profiles between plasma and serum samples, and only plasma-derived miRNAs were shown to be associated with clinical parameters. Similar results have been reported in other cancer types, indicating that miRNA expression in serum is not consistent with that in plasma and that higher miRNA concentrations are observed in serum samples than in plasma samples [39, 40]. Cell lysis and the presence of the “miRNA trafficking system” in cellular communication might be two plausible explanations for this discrepancy [41–43]. During the blood coagulation process, cell lysis may cause the release of miRNAs from vesicles in various cells and cell structures including platelets, circulating tumor cells, and exosomes to a certain extent. Meanwhile, blood cells are exposed to the stressful process of coagulation, which stimulates “miRNA trafficking system” to release certain miRNAs. This process does not occur during the isolation of plasma samples. The expression levels of serum-derived miRNAs were not correlated with clinical parameters in our study, probably because the miRNAs released during the coagulation process may have been irrelevant for BC. Unexpectedly, we observed a negative correlation between the expression levels of miRNAs (miR-106a-5p and miR-20b-5p) between plasma and serum, but the underlying mechanism remains unknown. Thus, whether miR-106a-5p and miR-20b-5p have specific association with the coagulation process still needs to be further clarified.

Notably, the overlapping miRNAs in plasma and serum were miR-106a-5p and miR-20b-5p, and the combination of the two miRNAs could accurately identify BC. Intriguingly, among the 6 identified miRNAs, the expression of only the two overlapping miRNAs was consistently significantly upregulated in BC tissues samples. Moreover, higher expression levels of plasma miR-106a-5p and miR-20b-5p were observed in patients with lower histological grade, ER-positive status, and HER2-negative status. Our findings indicated the significant roles of the two miRNAs in the initiation and progression of BC. In accordance with our findings, miR-106a from different sample sources has been reported to be upregulated in BC [18, 44–46]. miR-106a has been found to be significantly upregulated more than twofolds in BC samples of both tissues and matching sera compared with their controls [44], and miR-106a from plasma has been explored as a potential noninvasive biomarker for metastatic BC [45]. In addition, miR-106a has been revealed to be related to the ErbB signaling pathway [18, 47]. miR-106a is involved in a multitude of physiological and pathophysiological processes, including cell proliferation and apoptosis, invasion, metastasis, and drug resistance [48]. Among the multiple target genes of miR-106a in cancer cells, ZBTB4 is a transcriptional repressor regulating EZH2, a factor associated with decreased survival among BC patients [49]. miR-106a might thus have a potential role in BC pathogenesis and could be used as a subtype-specific biomarker for BC, but these data need to be further confirmed. To the best of our knowledge, this is the first report on the diagnostic value of miR-20b-5p in blood samples from BC patients. Two other studies focused on the prognostic role of miR-20b-5p using tissue samples but described inconsistent results [50, 51], while studies on miR-20b in other cancers have seemingly reached a consensus that high level of miR-20b expression is associated with poor prognosis [52–54]. miR-20b has been found to target tumor suppressors like PTEN and BRCA1 [55, 56], which could contribute to breast tumorigenesis. The biological roles of miR-20b in BC need to be investigated further.

In addition to the two overlapping miRNAs, the other identified miRNAs had also been studied earlier. Circulating miR-92a-3p was identified as a potential biomarker for BC in previous studies [16, 57, 58]. Moreover, miR-92a-2-5p and miR-19b-3p were discovered for the first time as valuable biomarkers of BC in our study, although these miRNAs had been previously reported in many cancer types [59–63]. miR-92a seems to be involved in E2F1 posttranscriptional regulation, which can cause both cell-cycle arrest and apoptosis [64]. In addition, miR-19 has been found to trigger epithelial–mesenchymal transition (EMT) by inhibiting the expression of PTEN [65]. As a paralogous cluster of miR-17–92, miR-106a–363 has also been reported to have an oncogenic potential in other malignancies, including human T-cell leukemia, cutaneous B-cell lymphoma, mantle cell lymphoma and Ewing sarcoma [14, 52, 66, 67]. These results supported our findings that the miR-106a–363 cluster might play an important role in the tumorigenesis of BC. on the one hand, and, on the other, suggested that the specificity of circulating miRNAs as potential biomarkers for BC is worthy of further research.

Based on the bioinformatics analysis in our study, the pathway “proteoglycans in cancer”, which affects the biology of various types of cancer, was found to be shared among the 6 miRNAs. In BC pathogenesis, altered expression of proteoglycans have strong effect on cell proliferation and differentiation [68]. Furthermore, accumulated knowledge indicates that proteoglycans play important roles in the breast tumor microenvironment [69]. Nevertheless, this pathway needs to be validated in the future. Recently, the identification of circulating miRNAs has been encouraged, and the understanding of their functions as well as the underlying mechanisms is warranted.

Exosomes are membrane-bound vesicles of endocytic origin that contain mRNAs, miRNAs, and small regulatory RNAs (sRNAs), and miRNA-containing exosomes are secreted into the circulation [70–73]. Exosomes may be extracted from various body fluids, including serum. Recent studies have shown that exosomal miRNAs in serum could partially be used to identify specific molecular subtypes of BC and could thus be promising prognostic markers for the metastatic progression in BC [74, 75]. We further evaluated plasma and serum exosomal miRNAs (miR-106a-3p, miR-106a-5p, miR-20b-5p, and miR-92a-2-5p in plasma and miR-106a-5p, miR-19b-3p, miR-20b-5p, and miR-92a-3p in serum) to explore their potential use as biomarkers for BC. The results were similar for plasma and serum, and indicated that compared to those in HCs, all miRNAs except for plasma-derived exosomal miR-20b-5p were significantly upregulated in BC patients. These exosomal miRNAs might be involved in the initiation and progression of BC and thus need to be investigated further. It has been revealed that circulating miRNAs are not only encapsulated in exosomes but that they can also be cofractionated with protein complexes for protection against plasma RNases, such as Argonaute2 complexes [76]. Therefore, we speculate that miR-20b-5p binds to Argonaute2 complexes in plasma. Future studies of miRNA biomarkers are needed using both exosomes and protein complexes.

Circulating miRNAs are a class of promising noninvasive biomarkers for cancer diagnosis due to their characteristics such as high stability, low complexity, and similarity in carrier profiles [22, 77]. However, the translation of the novel biomarkers into the clinic remains a work in progress. First, it is urgent to establish standardized procedures for sample processing and profiling, RNA isolation, data analysis, etc. Regarding the selection of endogenous control, the optimal reference varies with different types of cancer. Second, more function-based researches to study the circulating miRNA biology would help to identify them as reliable biomarkers [78]. At present, the miRNA-based test may not be able to screen for BC individually, but it can combine with current standard-of-care diagnostic tools, i.e., mammographic screening, which results in a gradual approach to clinical use.

## Conclusions

In our study, we identified four plasma miRNAs (miR-106a-3p, miR-106a-5p, miR-20b-5p, and miR-92a-2-5p) and four serum miRNAs (miR-106a-5p, miR-19b-3p, miR-20b-5p, and miR-92a-3p) from the miR-106a–363 cluster, and these could be used as novel noninvasive biomarkers for BC detection. The presence of several miRNAs associated with specific clinical parameters, especially the two overlapping miRNAs in plasma and serum (miR-106a-5p and miR-20b-5p), indicated the potential roles of these miRNAs in BC pathogenesis. Overall, our findings may contribute to improve the available diagnostic methods and promote the application of circulating miRNAs in the screening of BC patients.

## Electronic supplementary material

Below is the link to the electronic supplementary material.

Supplementary material 1 (DOC 1161 kb)

## Abbreviation

BC: breast cancer
HCs: healthy controls
ER: estrogen receptor
HER2: human epidermal growth factor receptor 2
miRNA: microRNA
PCR: polymerase chain reaction
qRT-PCR: quantitative reverse transcription polymerase chain reaction
ROC curve: receiver operating characteristic curve
AUC: area under the ROC curve
FC: fold change
