# Supplementary material for: Circulating microRNAs from the miR-106a–363 cluster on chromosome X as novel diagnostic biomarkers for breast cancer
Source: Breast Cancer Res Treat. 2018 Mar 20;170(2):257–70. doi: 10.1007/s10549-018-4757-3 (PMC5999170; doi:10.1007/s10549-018-4757-3)
Supplement: Supplementary file 1 — Supplementary material 1 (DOC 1161 kb) [file 10549_2018_4757_MOESM1_ESM.doc]

**Additional file 1:** Characteristics of individuals contributing the 400 plasma samples and 406 serum samples in three phases

| **Plasma**  **Characteristics** | **Training phase** | | | **Testing phase** | | | | | **External validation phase** | |
| --- | --- | --- | --- | --- | --- | --- | --- | --- | --- | --- |
|  | **BC patients** **(%)** | | **HCs (%)** | | | **BC patients (%)** | | **HCs (%)** | **BC patients (%)** | **HCs (%)** |
| **Number** | 24 | 24 | | | 146 | | 146 | | 30 | 30 |
| **Age at diagnosis**  **< 50**  **≥ 50** | 7 (29.2)  17 (70.8) | 10 (41.7)  14 (58.3) | | | 56 (38.4)  90 (61.6) | | 68 (46.6)  78 (53.4) | | 13 (43.3)  17 (56.7) | 10 (33)  20 (67) |
| **TNM stage**  In situ  I  II  III | 2 (8.3)  6 (25)  14 (58.3)  2 (8.3) |  | | | 9 (6.2)  41 (28.1)  66 (45.2)  30 (20.5) | |  | | 2 (6.7)  7 (23.3)  14 (46.7)  7 (23.3) |  |
| **Grade**  I  II  III | 2 (8.3)  8 (33.3)  14 (58.3) |  | | | 6 (4.1)  63 (43.2)  77 (52.7) | |  | | 1 (3.3)  13 (43.3)  16 (53.3) |  |
| **Epithelial subtype**  Luminal  HER2-enriched  Triple-negative  In situ | 10 (41.7)  6 (25)  6 (25)  2 (8.3) |  | | | 82 (56.2)  24 (16.4)  31 (21.2)  9 (6.2) | |  | | 15 (50)  6 (20)  7 (23.3)  2 (6.7) |  |

| **Serum**  **Characteristics** | **Training phase** | | **Testing phase** | | | **External validation phase** | | |
| --- | --- | --- | --- | --- | --- | --- | --- | --- |
|  | **BC patients** **(%)** | **HCs (%)** | | **BC patients (%)** | **HCs (%)** | **BC patients (%)** | **HCs (%)** |  |
| **Number** | 24 | 24 | | 150 | 148 | 30 | 30 | |
| **Age at diagnosis**  **< 50**  **≥ 50** | 11 (45.8)  13 (54.2) | 9 (37.5)  15 (62.5) | | 61 (40.7)  89 (59.3) | 73 (49.3)  75 (50.7) | 12 (40)  18 (60) | 14 (46.7)  16 (53.3) | |
| **TNM stage**  In situ  I  II  III | 2 (8.3)  5 (20.8)  12 (50)  5 (20.8) |  | | 8 (5.3)  45 (30)  76 (50.7)  21 (14) |  | 2 (6.7)  8 (26.7)  16 (53.3)  4 (13.3) |  | |
| **Grade**  I  II  III | 1 (4.2)  8 (33.3)  15 (62.5) |  | | 7 (4.7)  48 (32)  95 (63.3) |  | 1 (3.3)  10 (33.3)  19 (63.3) |  | |
| **Epithelial subtype**  Luminal  HER2-enriched  Triple-negative  In situ | 8 (33.3)  6 (25)  8 (33.3)  2 (8.3) |  | | 59 (39.3)  37 (24.7)  46 (30.7)  8 (5.3) |  | 13 (43.3)  6 (20)  9 (30)  2 (6.7) |  | |

**Additional file 2:** Stability of the expression of potential reference miRNAs (miR-16-5p, miR-1228-3p, miR-103a-3p, U6 and miR-191-5p) using GeNorm Version 3.5 (A: In plasma, B: In serum)


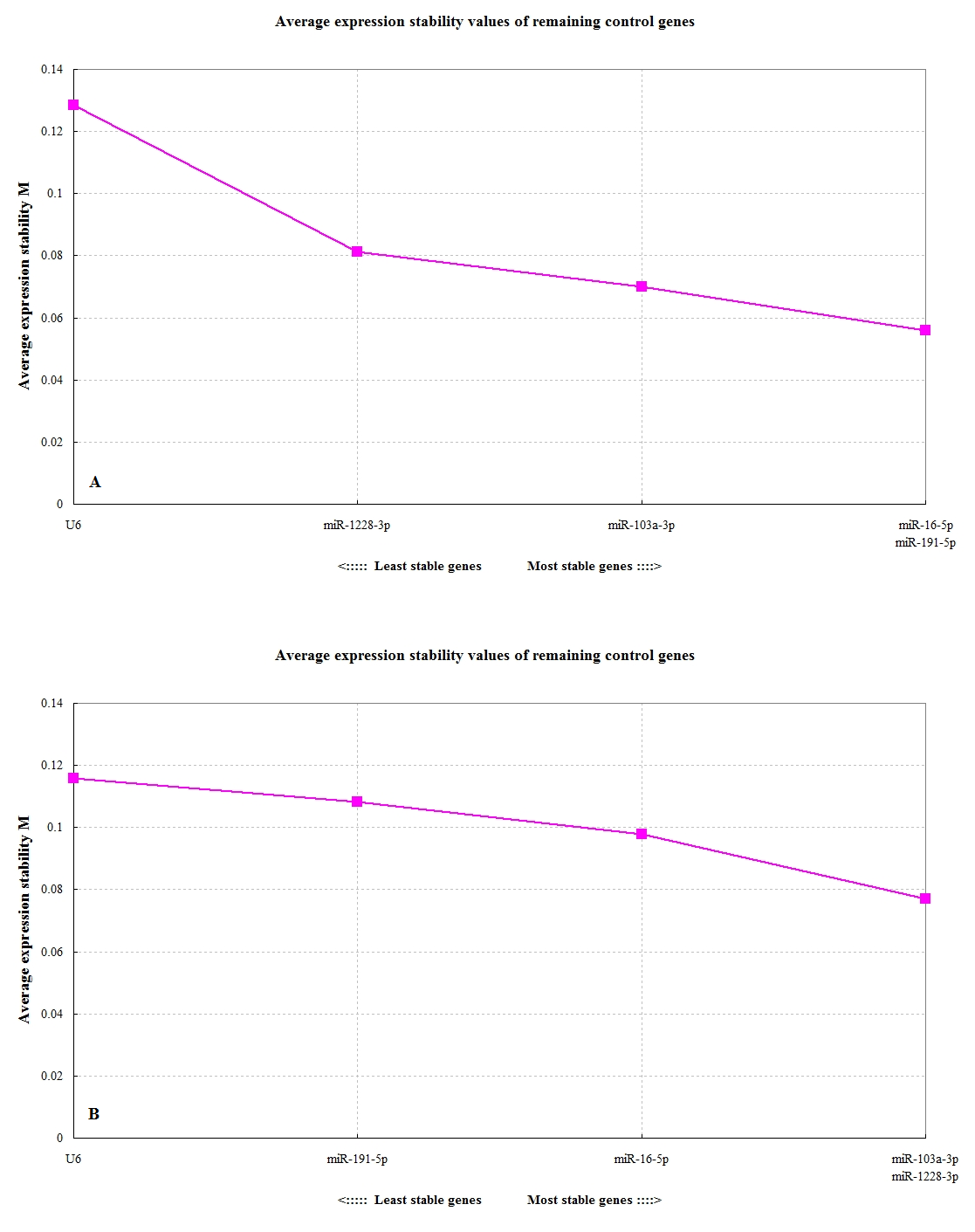


**Additional file 3:** Expression levels of the miRNAs not passed through the training phase (presented as mean ± SD; ΔCT, relative to combination of cel-miR-39 and miR-16 in plasma, cel-miR-39 and miR-1228 in serum; FC: fold change)

| **Plasma** | | | | | **Serum** | | | | |
| --- | --- | --- | --- | --- | --- | --- | --- | --- | --- |
| **miRNA** | **BC** | **HC** | **FC** | **P value** | **miRNA** | **BC** | **HC** | **FC** | **P value** |
| miR-363-3p | -1.83±3.03 | -1.33±1.56 | 1.41 | 0.934 | miR-363-3p | -8.46±1.68 | -8.08±1.97 | 1.31 | 0.650 |
| miR-19b-5p | -9.49±3.58 | -8.97±2.56 | 1.43 | 0.773 | miR-19b-5p | -16.42±1.53 | -16.16±1.38 | 1.20 | 0.503 |
| miR-20b-3p | -0.75±3.15 | -0.27±2.35 | 1.39 | 0.364 | miR-20b-3p | -7.55±1.29 | -7.08±1.55 | 1.38 | 0.284 |
| miR-19b-3p | -9.07±1.43 | -8.62±1.69 | 1.36 | 0.421 | miR-92a-5p | -8.71±1.87 | -8.44±1.86 | 1.21 | 0.550 |
| miR-18b-5p | -0.39±3.54 | 0.96±1.71 | 2.83 | 0.183 | miR-18b-5p | -4.98±2.01 | -4.69±1.78 | 1.22 | 0.567 |
| miR-363-5p | -2.21±3.59 | -1.86±1.79 | 1.27 | 0.665 | miR-106a-3p | -6.43±1.51 | -6.42±1.88 | 1.00 | 0.934 |
| miR-92a-3p | -10.62±1.58 | -10.70±1.09 | 0.95 | 0.386 | miR-363-5p | -7.49±2.06 | -7.87±2.07 | 0.77 | 0.456 |
| miR-18b-3p | -0.73±3.46 | 0.32±2.14 | 2.12 | 0.371 | miR-18b-3p | -6.25±1.66 | -5.31±1.95 | 1.92 | 0.060 |

**Additional file 4:** ROC curves of the identified miRNAs for discriminating BC patients from HCs in the two combined phases


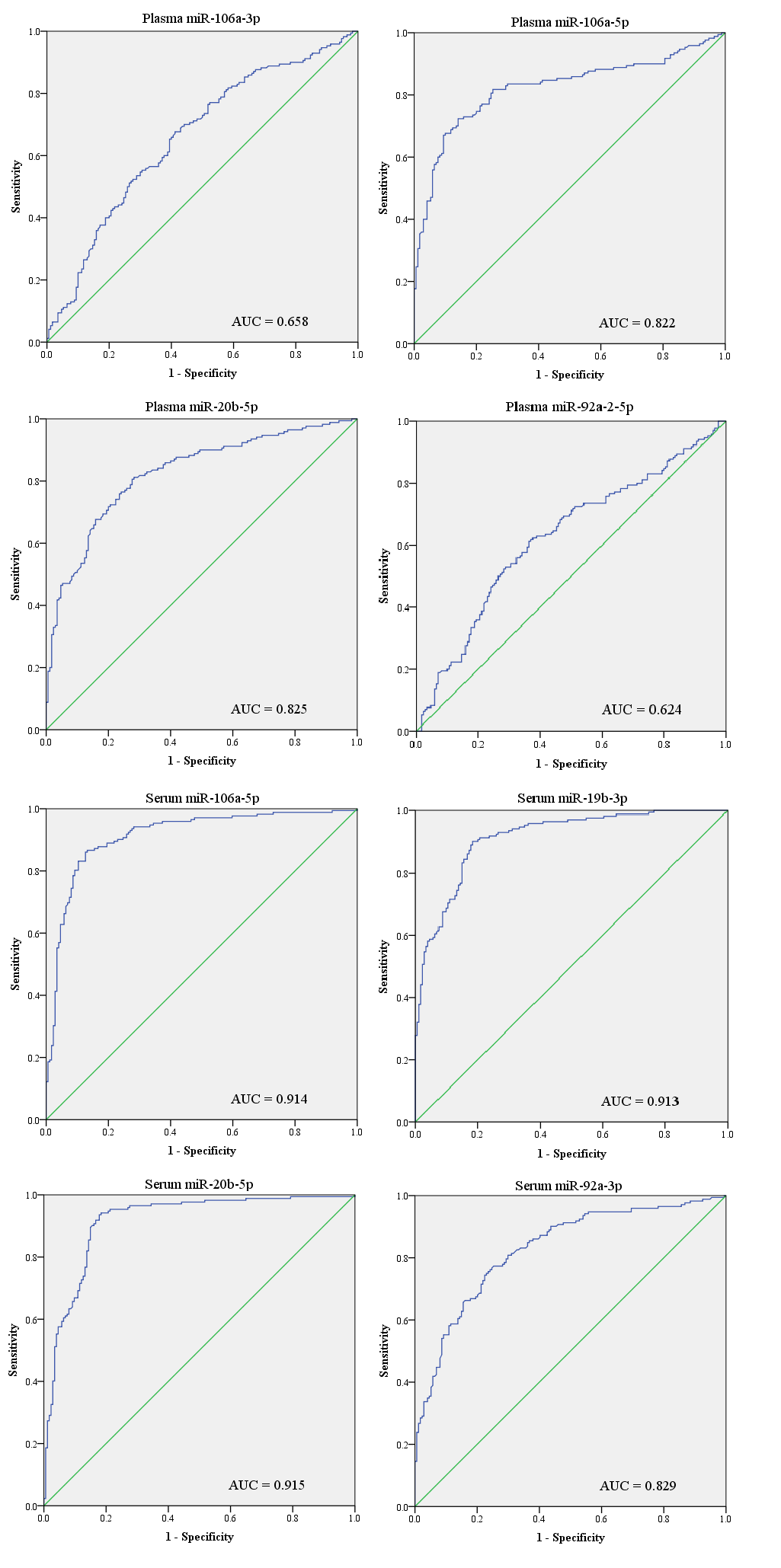


**Additional file 5:** Expression levels of the identified miRNAs in the external validation phase (presented as mean ± SD; ΔCT, relative to combination of cel-miR-39 and miR-16 in plasma, cel-miR-39 and miR-1228 in serum)

| **Plasma** | | | | | **Serum** | | | | |
| --- | --- | --- | --- | --- | --- | --- | --- | --- | --- |
| **miRNA** | **BC** | **HC** | **FC** | **P value** | **miRNA** | **BC** | **HC** | **FC** | **P value** |
| miR-106a-3p | -2.49 ± 1.78 | -0.76 ± 1.18 | 3.31 | <0.001 | miR-106a-5p | -13.99 ± 0.93 | -10.99 ± 1.09 | 7.97 | <0.001 |
| miR-106a-5p | -5.88 ± 1.18 | -4.71 ± 0.72 | 2.25 | <0.001 | miR-19b-3p | -15.20 ± 0.86 | -13.05 ± 1.19 | 4.42 | <0.001 |
| miR-20b-5p | -4.87 ± 0.87 | -3.86 ± 0.57 | 2.01 | <0.001 | miR-20b-5p | -13.56 ± 0.89 | -10.08 ± 1.28 | 11.16 | <0.001 |
| miR-92a-5p | 0.16 ±2.28 | 1.49 ±1.33 | 2.52 | 0.006 | miR-92a-3p | -17.17 ± 1.20 | -15.22 ± 1.25 | 3.84 | <0.001 |

**Additional file 6:** Correlation between the relative expression of the miRNA in plasma and in serum (A. miR-106a-5p; B. miR-20b-5p; X-axes: -ΔCT of miRNAs in plasma; Y-axes: -ΔCT of miRNAs in serum)

**
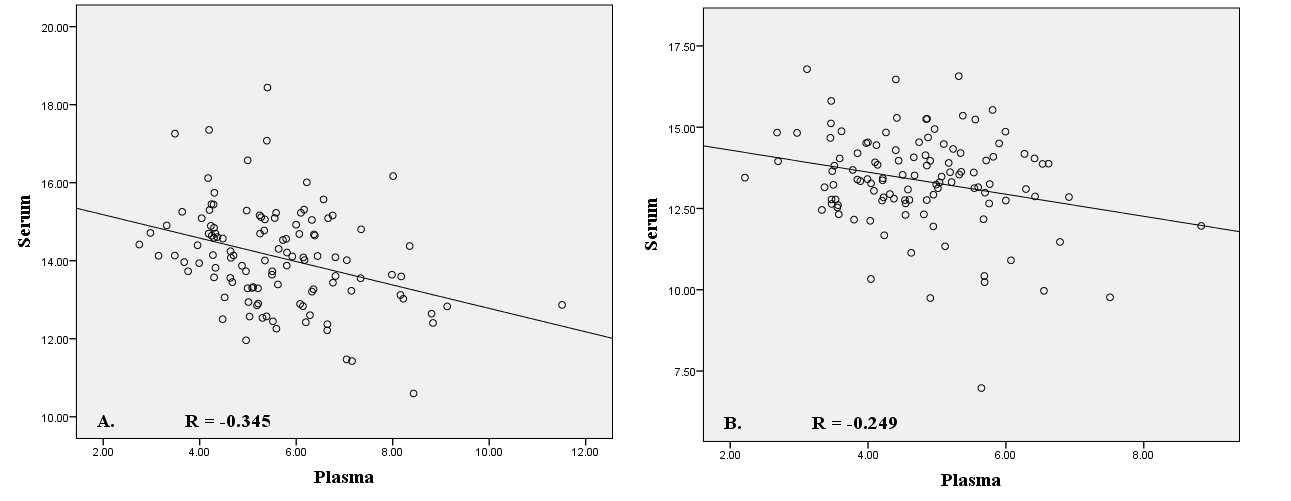
**

**Additional file 7:** Association of the identified plasma miRNAs with different clinicopathological parameters (histological grade, ER and HER2 status). Y axis represents the relative expression (2-ΔΔCt). Horizontal line: mean with SEM. N: normal controls; *P<0.05


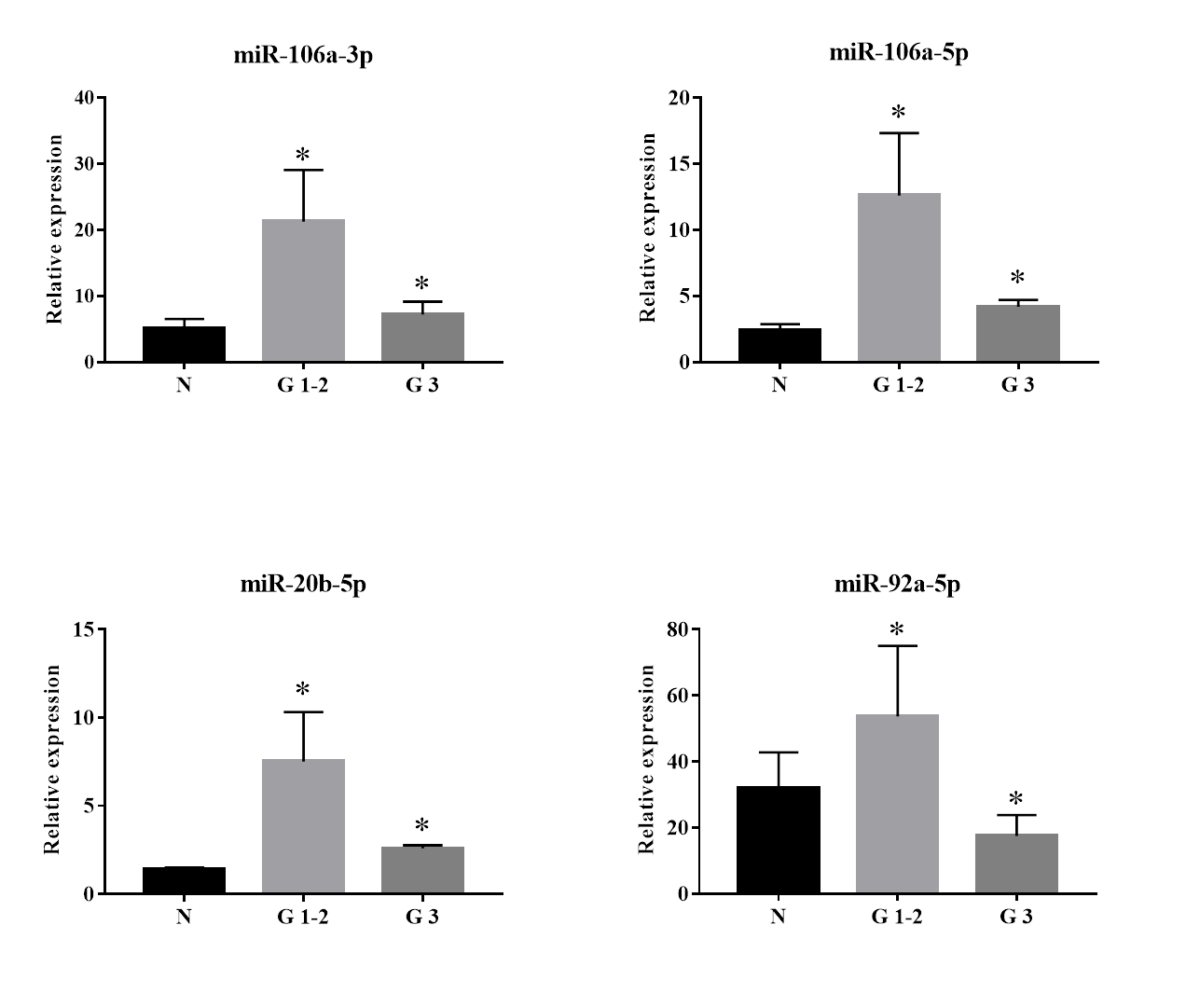


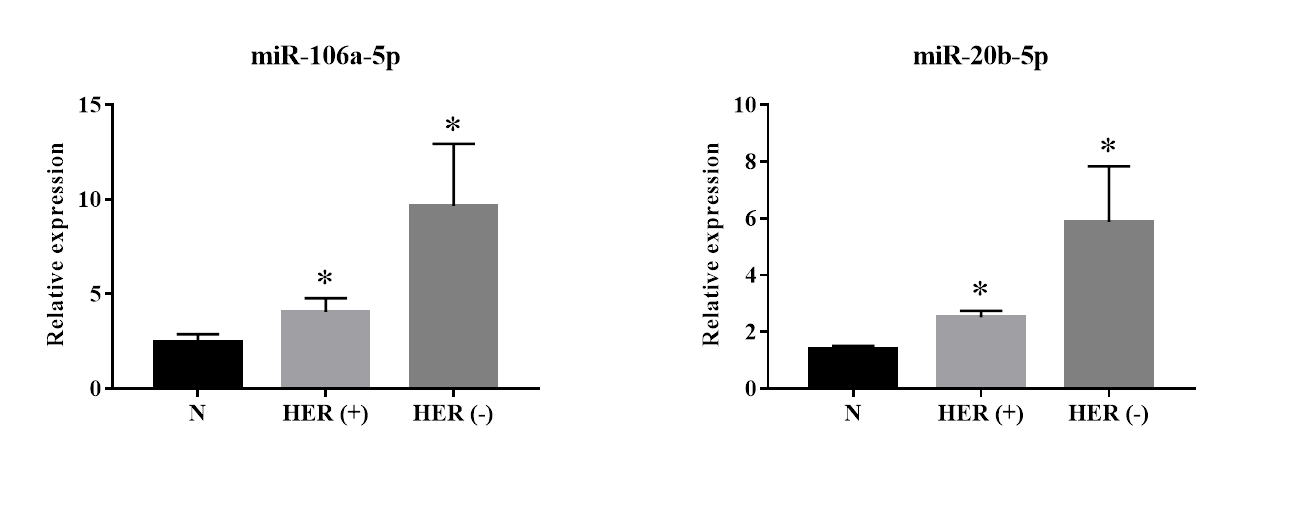

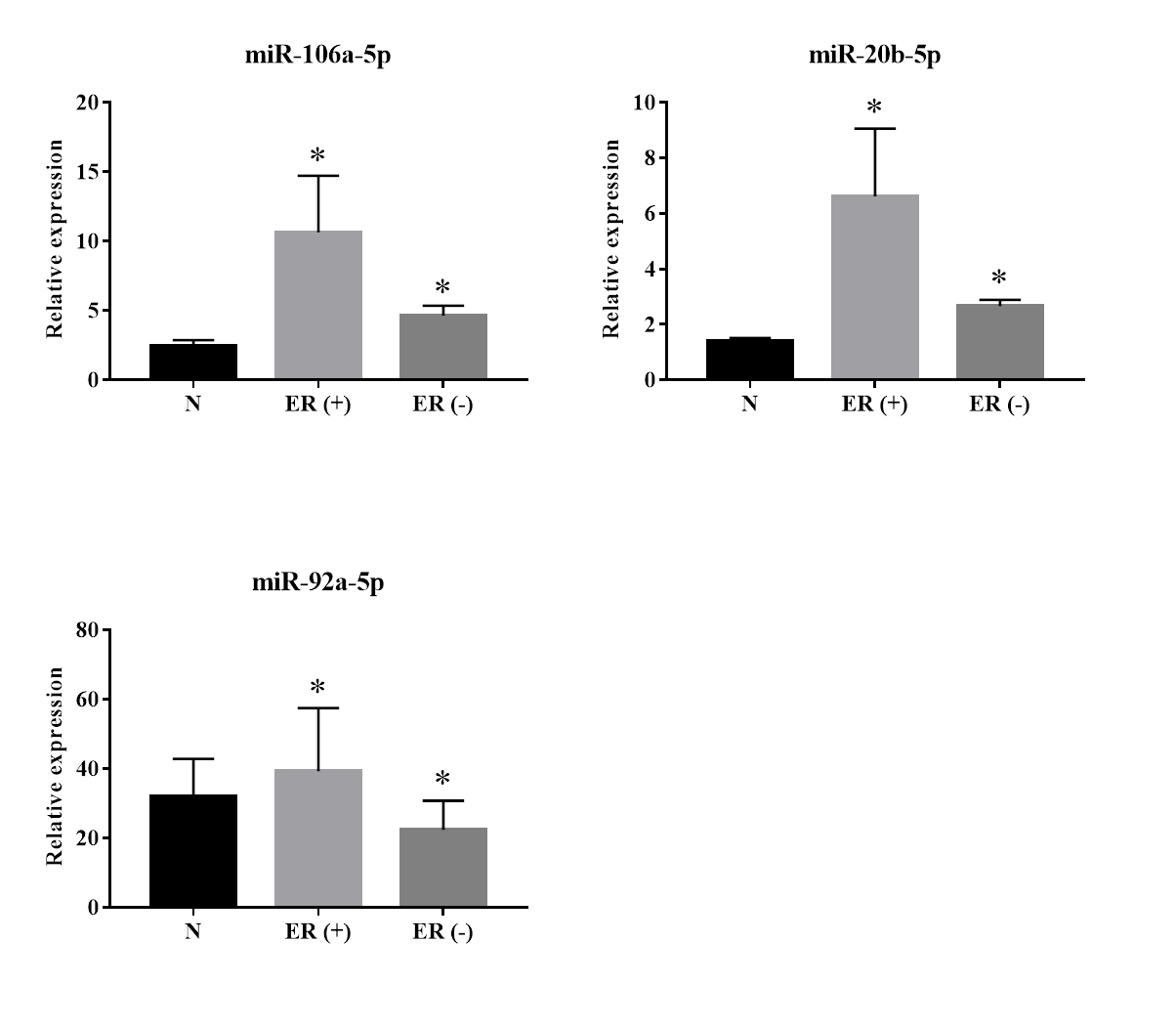


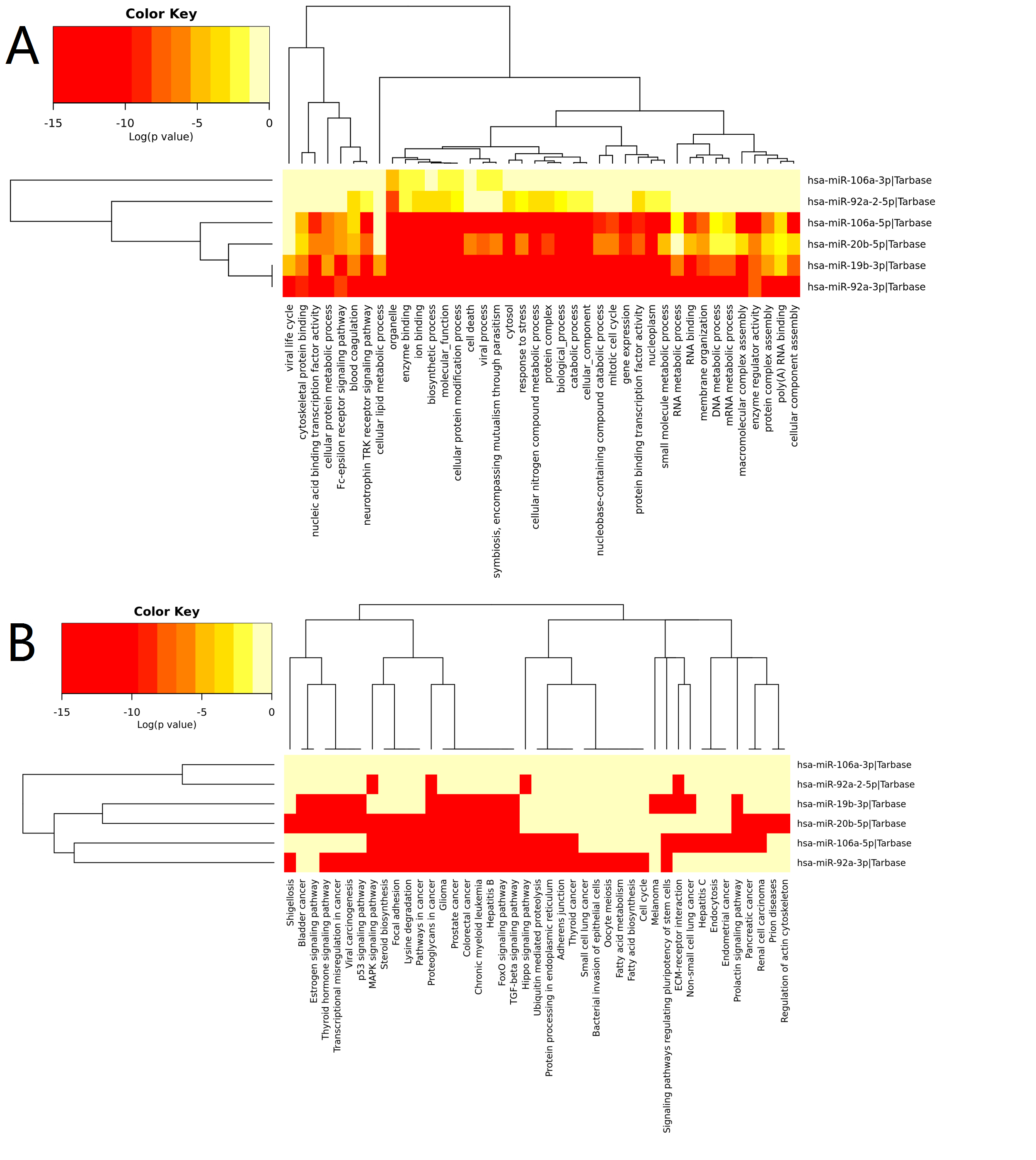
**Additional file 9:** Heatmaps of targeted pathways of the identified miRNAs using GO category analysis (A) and KEGG pathway analysis (B)
